# Optimizing antimicrobial prescription through e-health: setting, dosing, timing and stewardship

**DOI:** 10.1186/2047-2994-4-S1-O3

**Published:** 2015-06-16

**Authors:** C Palos, A Bispo, P Rodrigues, L França, M Capoulas

**Affiliations:** 1 Hospital Beatriz Ângelo Loures Portugal

## Introduction

Antimicrobial resistance is a major issue of of healthcare and new resistances are being observed. Antibiotics should be used strictly . When prescribed, they should be selected in accordance to a diagnosis, epidemiology and other factors, requiring appropriate dosing and duration of treatment. Antibiotic prescription should be seen as a team decision, where antimicrobial stewardship plays a major role. E-health, defined as the intensive use of information and communication technologies in the health sector, should be maximized in order to help physicians in prescribing right antibiotics on the right contexts, dosing and duration.

## Objectives

To optimize global antimicrobial prescription in a paper free hospital.

## Methods

A new template for antimicrobial prescription was created, allowing visualization and printing of first line guidelines. When prescribing antimicrobials, context is firstly requested: surgical prophylaxis, therapeutics, others (droplists for all). In doing so, the usual dose and the maximum duration for the selected context automatically appear by default (1 day for SSI prophylaxis; variable days for each infection, 7 days for the majority). When the selected antimicrobial is not matched and/or it belongs to a group of conditioned use (quinolones, carbapenems, vancomycin, e.g.), a pop-up message appears on the screen and justification is mandatory. An automated e-mail with those details is immediately sent to a subgroup of the ICAC and Pharmacy.

## Results

Antimicrobial prescriptions are conditioned to the clinical setting. Dose and duration of antimicrobial therapy is made adequate. ICAC and Pharmacy provide real time antimicrobial stewardship. Inadequate use of antibiotics such as quinolones and carbapenems is limited to adequate contexts. Data analysis allows feedback to prescribers.

## Conclusion

E-health is a major tool in order to improve quality and safety of antimicrobial use, thus minimizing the emergence of resistant bacteria through improvement on prescription, antimicrobial stewardship and data analysis.

## Disclosure of interest

None declared.

